# Physiological Action of Yerba Santa

**Published:** 1882-04-20

**Authors:** 


### PHYSIOLOGICAL ACTION OF YERBA SANTA.

Dr. Briggs: Mr. Chairman, I have a few notes of a case involving the physiological action of yerba santa. It is not a very clear and definite case; still there are some important facts connected with it, and as yerba santa is a somewhat new remedy, we may as well make note of them. A patient whom I have seen from time to time in the last two years—a stout woman 50 years of age, sanguine, plethoric—happening to be suffering from some cough, went to an apothecary store, and speaking to the clerk, he very kindly gave her a bottle containing the extract of yerba santa. The prescription was:

| | | |
|---|---|---|
| ℞ | Ext. of yerba santa | ʒ iv. |
| | Syr. tolu | ℥ iss. |
| | Liquor potass | gtt. xv. |

She took only a few tastes of the bottle when she received it. But on the night of the 20th of January, on going to bed, her cough worried her very much, and as the bottle was nearly full, she thought to make sure she had better take a good deal of it, and she took at one draught all the bottle contained.

Dr. Pollak: Did she take it all?

Dr. Briggs: Yes, sir. There were two or three drachms of extract of yerba santa in the bottle, and she took it all. This was to relieve the cough, it must be remembered. At half past 5 o'clock in the morning, a woman who was sleeping in the same room with her got up, and this person observed her turning over and concluded she was awake and going to get up at once.

The room-mate arose, dressed herself, and went out. At 7 o'clock she returned, and found the patient lying in a heap near the end of the room, in a very constrained posture, her head bent down with a sharp twist of the neck.

There was a flood of urine on the carpet, and also in the bed. She was in a house where they are accustomed to take care of the sick, and the alarm being given, she was found to be without breath or pulse. They raised her, opened her mouth, forced some brandy down her throat, and put her feet in hot mustard water. The treatment was followed, almost immediately, by a return of the pulse. If she had not been discovered, she would have died very quickly indeed. In 30 minutes she had recovered sufficiently to answer "yes" and "no." I saw her about 8 o'clock in the morning, and at that time, you will observe, brandy had been administered to her and the mustard application made, and she had been put back to bed and was warm. I found her face red. There was no convulsive action. Her mouth was firmly set, but it was not twisted to one side. When I spoke to her, she answered me almost at once, she knowing my voice. She had a full pulse then; it was 80; there had been no vomiting. Judging from this strong pulse, red face, and relapses into stupidity, that there was some congestion of the brain, I gave at intervals two bread pills containing a drop of croton oil each, and also a mustard enema. They told me she would partially recover consciousness and speak to them, and then she would relapse into unconsciousness with the firm setting of the mouth. In falling she had injured her eye and bruised her shoulder, which kept her confined for a little while, but she recovered very kindly indeed. She could talk connectedly by 10 or 11 A. M. She remembered that she had taken the medicine, and that she had a desire to urinate. After that came a blank, until she revived after the brandy, and was conscious of her friends about her. It was probable that the taking of the drug was followed by sleep and stupor. The desire to urinate was so strong that it partially roused her. She did not remember getting up, and probably did so almost automatically, beginning to discharge urine before she left the bed. The bladder seems to have been very full, perhaps owing to the drug.

She got out of bed, and had fallen in consequence of the loss of nervous power occasioned by the action of the drug; then the constrained position in which she fell, combined with the action of the cold—for the weather was very cold—stopped the functions of the heart and lungs, and would have caused her death if it had not been for the timely aid she received. The redness of her face may have been from the drug, or may have been from the brandy only. The record then is, that she took three drachms of extract of yerba santa and recovered.

Dr. Ford: This prescription was used as an anti-spasmodic, was it not, to relieve cough?

Dr. Briggs: The idea was that it would reduce the irritation of the mucous membrane. I think that was the theory the clerk acted on in giving the prescription.—Proceedings St. Louis Medical Society.—*St. Louis Medical and Surgical Journal*, April, 1881.

---

**Astringent Cotton Tampons,** saturated with a solution of glycerine, alum and carbolic acid, are recommended in treatment of uterine displacements, in place of pessaries, stems, etc.—*Monthly Review.*

